# In vitro evaluation of a novel self-adhesive composite primer modified with nano zinc oxide and hydroxyapatite: antibacterial activity, marginal microleakage, and interfacial elemental analysis

**DOI:** 10.1186/s12903-026-09627-2

**Published:** 2026-08-27

**Authors:** Raghda Kamh, Alshaymaa Abdelmonaem Mohammed, Hamdy Mahmoud Badreldin, Esraa Abdel Ghaffar El Gezawy

**Affiliations:** 1 Faculty of Oral and Dental Medicine, Egyptian Russian University, Cairo, Egypt; 2 Basic and Medical Sciences Department, Faculty of Oral and Dental Medicine, Egyptian Russian University, Cairo, Egypt; 3 Faculty of Dental Medicine - Boys- Cairo, Al Azhar University, Cairo, Egypt; 4 Faculty of Dentistry, Galala University, Suez, Egypt

**Keywords:** Self-adhesive resin composite, Zinc Oxide (ZnO), Hydroxyapatite (HA), Nanoparticles, *Streptococcus mutans*, Dental microleakage, Spectrometry, X-ray Emission (EDX)

## Abstract

**Background:**

This study aimed to investigate the antibacterial effects of a self-adhesive 10-MDP contained Primer (Stela primer) modified with nano-sized zinc oxide (ZnO) and hydroxyapatite (HA) particles. Furthermore, it evaluated the extent of marginal microleakage in Class II restorations and conducted elemental analysis of the tooth–primer interface using energy-dispersive X-ray spectroscopy (EDX).

**Materials and methods:**

The sixty-three premolars enrolled in this study were subjected to class II cavity preparation and divided into three groups according to the primer used (modified Stela primer containing zinc oxide nanoparticles (ZnO-NPs), hydroxyapatite nanoparticles (HA-NPs), and Stela primer without modification). Stela self-adhesive resin composite was applied to all teeth. The teeth were subjected to a microleakage test (*n* = 21) for each group and elemental analysis of the tooth–primer interface using EDX (*n* = 3) for each group. All modified and non-modified primers were evaluated for their antibacterial activity against *Streptococcus mutans (S. mutans).*

**Results:**

No statistically significant differences in marginal microleakage were observed between the modified and unmodified groups at either the occlusal or gingival margins (*P* = 0.34 gingivally; *P* = 0.15 occlusally). EDX analysis showed a notable increase in calcium and phosphorus deposition at the tooth–primer interface in both nanoparticle-modified groups, especially in the HA-NP group, indicating interfacial mineralization (*P* < 0.001). The unmodified primer showed no significant changes in mineral content. Adding ZnO-NPs and HA-NPs to the Stela primer greatly improved its antibacterial effect against *Streptococcus mutans*, with HA-NPs producing the largest inhibition zone.

**Conclusions:**

Incorporating ZnO-NPs and HA-NPs into Stela primer enhances its biological potential; however, they did not significantly alter the sealing ability (microleakage) compared to the control. Both nanoparticle-modified primers exhibited potent and comparable bactericidal activity against *Streptococcus mutans*. At the same time, nano-hydroxyapatite demonstrated the most pronounced antibacterial effect and the most significant improvement in mineral deposition at the tooth–primer interface.

## Background

Resin composite restorations have emerged as the preferred treatment choice for patients and dentists worldwide, owing to their exceptional aesthetic qualities, mercury-free formulations, and minimally invasive restorative methods, with approximately 500 million composite restorations administered each year [1].

The characteristics of dental restorative materials present several difficulties, and finding an ideal material remains a current area of research. One of these challenges is that they do not bond directly to dental hard tissues, necessitating an adhesive coating for optimal adhesion, a light-curing unit, and consideration of polymerization shrinkage [2]. Adhesive failure at the dentin-composite interface is highly prevalent; in certain restorative dentistry studies, over 50% of failures are attributed to adhesive deficiencies, particularly in high-stress posterior areas [3, 4]. Although resin-based restorative materials may promote biofilm accumulation because of their surface characteristics and aging-related degradation, plaque accumulation alone is insufficient to initiate secondary caries. The development of secondary caries is a multifactorial process involving biofilm maturation, marginal degradation, bacterial penetration through defective tooth–restoration interfaces, dietary carbohydrate exposure, and host-related factors [5]. Degradation of the adhesive interface over time may facilitate bacterial infiltration and compromise the longevity of restorations, making secondary caries one of the leading causes of restoration replacement [6]. As a result, secondary caries is considered the primary cause of early failure, often necessitating replacement within 5 to 7 years. This can lead to more extensive and less durable restorations [7]. To overcome these clinical challenges, recent developments in restorative materials have focused not only on improving adhesive durability but also on optimizing polymerization mechanisms to reduce shrinkage stress and enhance marginal adaptation. Numerous curing methods have been developed to enhance the polymerization dynamics of clinical dental resin composites. These methods include self-curing, light-curing, and dual-curing composites. Chemically cured (self-cured) composite materials are utilized as direct restorative materials because of their minimal shrinkage stress, extended pre-gel phase, and gradual polymerization. Additionally, these materials allow for an unlimited depth of cure [8].

A self-cured, bulk-fill restorative material has recently been launched on the market (Stela, SDI, Victoria, Australia). Its material characteristics make it a promising option for primary molars, particularly in broad and shallow cavities, where it can provide a more consistent marginal seal and reduce the risk of leakage and postoperative sensitivity. Furthermore, the simplified two-step procedure (primer followed by bulk placement) shortens clinical time, an essential advantage in pediatric dentistry. Although evidence specific to primary dentition remains limited, Stela’s streamlined application and favorable mechanical performance suggest it may be an appropriate alternative to commonly used restorative materials in primary teeth, such as resin-modified glass ionomer and conventional resin composite [9, 10].

Stela polymerization began upon contact with the Stela primer, thereby enhancing the reaction at the tooth/primer interface. This approach uses an innovative primer devoid of tertiary amines. It incorporates glycerol dimethacrylate (GDMA), which may enhance polymerization, improve mechanical properties, increase adherence to dentin, and reduce water absorption and solubility [9].

Self-curing restorative materials exhibit reduced polymerization shrinkage compared to light-curing materials, attributable to their superior degree of conversion, reducing microleakage [2]. Microleakage is a significant factor in the failure of posterior restorations and warrants further study to enhance their longevity [11].

On the other hand, Stela™ Primer (SDI Ltd., Australia) contains the functional acidic monomer 10-methacryloyloxydecyl dihydrogen phosphate (10-MDP), which enables simultaneous demineralization [12] and the capacity to chemically bond with hydroxyapatite, resulting in the formation of stable, water-insoluble MDP-calcium salts that strengthen the hybrid [13]. These stable MDP–calcium salts contribute to durable chemical adhesion, nano-layering at the adhesive interface with enhanced hydrolytic stability and long-term bond durability [14, 15].

*Streptococcus mutans* and other oral bacteria proliferate in the microleakage space surrounding the restoration [16]. Functional monomers like MDPB, a derivative of alkylpyridinium halide and a member of the quaternary ammonium compound family noted for its broad-spectrum antimicrobial action, are essential to the antibacterial-based adhesives [17]. Recent research in restorative materials emphasizes the incorporation of nanoparticles and nanotubes to improve antibacterial and mechanical properties in dental materials [7]. Zinc oxide nanoparticles possess broad-spectrum antibacterial properties and have been incorporated into dental materials to inhibit bacterial colonization and reduce biofilm formation [18, 19]. Conversely, hydroxyapatite nanoparticles (HA-NPs) are bioactive calcium phosphate materials capable of promoting mineral deposition and supporting remineralization at the tooth–restoration interface [20]. Elemental analysis of the tooth/primer interfacial layer was done using an energy dispersive X-ray (EDX) to evaluate the change in mineral content after incorporation of HA-NPs [21].

This study was designed to investigate the antibacterial potential of Stela primer modified with nano-sized zinc oxide (ZnO) and hydroxyapatite (HA) particles. Furthermore, it evaluated the extent of marginal microleakage in Class II restorations and conducted elemental analysis of the tooth–primer interface using energy-dispersive X-ray spectroscopy (EDX). The null hypothesis was that incorporating nano-sized zinc oxide (ZnO) and hydroxyapatite (HA) particles into Stela primer would not result in any significant differences in antibacterial activity against *S. mutans*, marginal microleakage, or changes in mineral content compared to the unmodified primer.

## Materials and methods

### Materials

Materials used in this study are listed in Table 1.

**Table 1:** Composition of the restorative materials used in this study

Product name	Type	Composition	Product number	Manufacturer
STELA (SDI STELA Automix)	self-adhesive resin-based bulk-fill material	Catalyst: barium-glass, glass, ytterbium trifluoride (YbF3), silica, urethanedimethacrylate, initiators, stabilizers Base: strontium fluoroaluminosilicate glass, ytterbium trifluoride agglomerates (YbF3), silica, calcium aluminate (Al2CaO), urethanedimethacrylate, initiators, stabilizers	REF: 8,640,002 LOT: 1239063A	SDI Limited, Australia
STELA Primer		10-MDP, dimethacrylates, methyl ethyl ketone (MEK), water, initiators, stabilizers	REF: 8,640,006 LOT: 1,242,636	SDI Limited, Australia
Zinc Oxide nanoparticles	Powder	White to yellow powder, spherical-like shape nanoparticles with an average size of 20 ± 5 nm, and a molecular weight of 81.408 gm/mol		Nanogate Company- Cairo- Egypt
Hydroxyapatite nanoparticles	Powder	White powder, needle-like shape nanoparticles with an average size of width 10 ± 5 nm and length 100 nm ± 10 nm		Nanogate Company- Cairo- Egypt

### Research ethical approval

The study was approved by the Research Ethical Committee of the Faculty of Oral & Dental Medicine, Egyptian Russian University, research approval number [22]. The study was conducted in accordance with the ethical principles of the Declaration of Helsinki (2024 revision).

### Study design

**Fig. 1:**
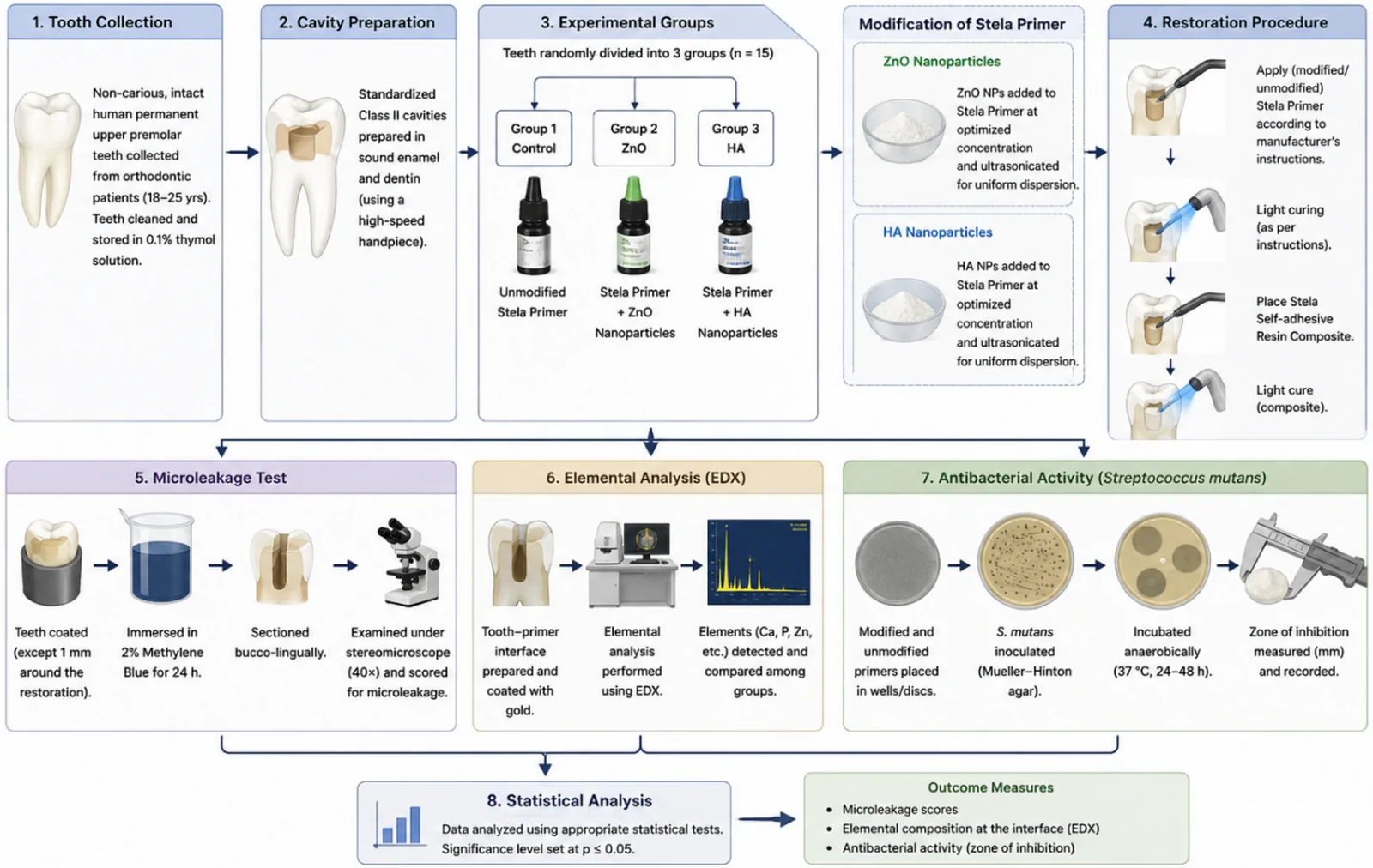
Experimental design and workflow of the study (Schematic illustration of the experimental protocol showing tooth collection, standardized Class II cavity preparation, random allocation of specimens, tested materials, followed by evaluation of antibacterial activity, marginal microleakage, and interfacial elemental analysis)

Non-carious, intact human permanent upper premolar teeth were collected from patients due to orthodontic treatment of a varied age range between 18 and 25 years. Standard class II cavities were created in sound enamel and dentin. The teeth were then divided into three groups and restored with modified Stela primer containing ZnO nanoparticles or HA nanoparticles, or with unmodified Stela primer. Stela self-adhesive resin composite was applied to all teeth according to the manufacturer's instructions. The teeth were subjected to a microleakage test and elemental analysis of the tooth–primer interface using EDX. All modified and non-modified primers were evaluated for their antibacterial activity against *Streptococcus mutans* (Fig. 1).

### Sample size calculation

The Sample size calculation was done based on effect size parameters derived from Teymoornezhad et al. (2016), who reported a 30% difference in microleakage score (0) between experimental and control groups in primary molars. Using a power of 80% and a 5% significance level, we will need to study 21 premolars per group and 3 for elemental analysis per group. Sample size was achieved using PS: Power and Sample Size Calculation Software Version 3.1.2 (Vanderbilt University, Nashville, Tennessee, USA [21, 23].

### Preparation of nano ZnO powder

ZnO nanoparticles were synthesized as detailed by Beek et al. [24] and Weller et al. [25] via the hydrolysis and condensation of zinc acetate dehydrate (Loba-Chemie, India) using potassium hydroxide (Merck, Germany) in an alcoholic medium (Merck, Germany) under low temperature conditions. The ZnO nanoparticles were allowed to settle at the bottom, after which the excess mother liquor was carefully removed, and the precipitate underwent washing with methanol. The precipitate was subsequently dispersed in a methanol-chloroform mixture [26, 27].

### Preparation of nano HA powder

Hydroxyapatite nanoparticles were synthesized using the wet-chemical method [18] with slight modifications. A solution of calcium chloride in water was stirred vigorously at room temperature. A solution of ammonium hydrogen phosphate was meticulously added dropwise to the calcium chloride solution. Ammonia solution was introduced gradually to modify the pH to 10. Following the reaction at 40 °C for four hours, the mixture was stirred for an additional 16 h at room temperature. HA-NPs precipitated as a white precipitate.

$$10\ \mathrm{Ca}\left(\mathrm{OH}\right)_2+6\mathrm{H}_3\mathrm{PO}_4\rightarrow\mathrm{Ca}_{10}\left(\mathrm{PO}_4\right)_6\left(\mathrm{OH}_2\right)+18\mathrm{H}_2\mathrm{O}$$

### Incorporation of ZnO and HA nanoparticles into Stela primer

ZnO and HA nano powder were weighed out on a sensitive balance (WTC 200, RADWAG®, Poland) and dispersed in Stela primer with a concentration 5% w/w (each nano powder was added in Stela primer individually in separate containers). To obtain a homogeneous phase, the mixture was sonicated for 30 min using an ultrasonic processor with 50 W input power (VCX 500, Sonics, USA) for 5 min, followed by vortexing for 10 min.

### Morphological examination of the prepared ZnO and HA nanoparticles in Stela primer

This study characterized ZnO and HA nanoparticles using a JEOL JEM-2100 high-resolution transmission electron microscope (TEM) operated at 200 kV. Samples for TEM were prepared by depositing a droplet of colloid suspension in the appropriate solvent onto a Formvar-coated, 300-mesh copper grid (Ted Pella) and permitting it to evaporate in air under ambient conditions. The size distribution and average size were assessed using image analysis software.

### Chemical analysis of ZnO and HA nanoparticles using X-Ray diffraction (XRD)

An XRD pattern has been obtained using the XPERT-PRO Powder Diffractometer system, with 2Theta (20°—80°), a Minimum step size of 2Theta: 0.001, and a wavelength (Ka) of 1.54614°.

### Sample preparation

Non-carious, intact human permanent upper premolar teeth were obtained from individuals aged 18 to 25 years undergoing orthodontic therapy. All individuals provided informed consent before extraction. The teeth were scaled and cleaned with tap water for surface debridement, then polished with a rubber cup and fluoride-free pumice. The specimens were preserved in distilled water at ambient temperature until utilized in the study [28]. Teeth had been extracted within the past three months. The teeth were visually inspected, and any fractures, cracks, previous restorations, or caries were excluded [29]. Standard class II cavities (occlusally and proximally) were created in sound enamel and dentin with a 90° cavo-surface angle (4 mm mesio-distal width, 3 mm buccolingual width, 2 mm pulpal floor depth). Moreover, the proximal cavity was drilled to a depth of one millimeter above the cement-enamel junction [7] with a standard variation of ± 0.2 mm; all dimensions were measured using a UNC Periodontal probe (SedraDent, Pakistan). The cavities were created using a high-speed air turbine handpiece that was equipped with a fissure diamond bur (size 2, Apple Dental, China). Subsequently, the burs were fine-ground with diamonds (FG-105, Azdent, China) while the water was continuously irrigated. Before restoration procedures, the specimens were soaked in distilled water maintained at 37 °C (pH 6.7) for up to 1 h [30].

### Samples grouping

According to the sample size, 63 premolars were used for the microleakage test, and 9 were subjected to EDX for elemental analysis of the tooth–primer interface. According to the manufacturer's instructions, the primer type used, the teeth subjected to the microleakage test were randomly divided into three groups (*n* = 21). Group M1: teeth restored with modified Stela primer with ZnO nanoparticles. Group M2: teeth restored with modified Stela primer with HA nanoparticles. Group M3: teeth restored with Stela primer without modification. The teeth subjected to the elemental analysis of the tooth–primer interface using EDX were randomly divided into three groups (*n* = 3). Group E1: teeth restored with modified Stela primer with ZnO nanoparticles. Group E2: teeth restored with modified Stela primer with HA nanoparticles. Group E3: teeth restored with Stela primer without modification.

### Restorative procedure

According to the manufacturer's instructions, both primer and resin composite were applied [30].

#### Application of Stela primer

Modified and non-modified primers were applied onto the prepared class II cavity surfaces and left for 5 s. The primer was gently air-dried until there was no movement of the primer with approximately (2–3 s) duration. The Stela primer began to cure upon contact with the Stela restorative material.

#### Application of Stela resin composite (Automix)

Paste was extruded into the cavity as a single increment (up to 5 mm thick). Air was avoided to prevent being trapped during the restoration. Care was taken to ensure the primer was in good contact with the restoration, as the composite self-cures within 4 min.

Immediately following the tooth restoration operation, the teeth were immersed in distilled water at 37 ± 2 °C in an incubator for 24 h and subsequently polished with abrasive discs (Sof-lex, 3 M ESPE, St. Paul, MN, USA).

### Samples preparation for the microleakage test

The restored premolars selected for the microleakage test were thermocycled (SD Mechatronic Thermocycler, Germany) for 1,000 cycles in a water bath, alternating between 5 °C and 55 °C. Following thermocycling, the apex of each tooth was sealed with wax, and the entire surface was coated with two layers of nail varnish. All teeth were submerged in a 0.1% methylene blue solution for 24 h at ambient temperature, subsequently extracted, and thoroughly washed under running water. They were then sectioned mesiodistally. Dye penetration was evaluated at 50X magnification using a stereo optical microscope (XCAM 1080P8MPB, ToupTek Photonics, Zhejiang, China) [11].

### Quantitative measurement of microleakage

Image analysis software (ImageView Analysis, ToupTek Photonics, Zhejiang, China) was linked to a stereo optical microscope for precise quantitative analysis. Microleakage length, in microns, gingivally and occlusally, was measured, denoting the actual linear distance the tracer dye has penetrated along the tooth-restoration interface. These numerical results were then used to perform statistical comparisons between groups [28].

Two Stereo microscope images were selected as representative for each group, showing the cross-section of the prepared sample, the surface morphology, and the layer interface, with the measured microleakage length indicated.

### Elemental analysis of tooth/primer interface using energy dispersive X-ray (EDX)

Specimens were prepared for energy-dispersive X-ray analysis by longitudinally sectioning them into two halves in the mesiodistal plane using an automated diamond saw (Isomet 4000, Buehler, Germany), with abundant water coolant employed to obtain the two halves. Subsequently, specimens were manually polished using #600 silicon carbide sandpaper under flowing water and subsequently subjected to ultrasonic cleaning. Samples were prepared by immersion in HCl for 5 s, then washed away with distilled water, followed by immersion in diluted NaOCl (1:3) for 10 min, and then washed with distilled water. Cylindrical plastic molds were custom-manufactured using plastic syringes and later filled with self-curing acrylic resin (Acrostone, Anglo-Egyptian Company, Cairo, Egypt). Each specimen half was fixed in resin with the restoration and dentin fronting upward, and aligned parallel to the horizontal plane of the acrylic resin. Specimens were preserved by placing a moist, lint-free laboratory napkin on the examined surface and removing it shortly prior to testing to prevent dehydration [21].

Morphology of the prepared samples was investigated using TESCAN VEGA COMPACT SEM with a Tungsten Filament as the electron source and an attached EDX detector. Elemental weight% of Calcium and Phosphorus was detected at two areas: first, away from the restoration, and second, at the tooth/primer interface. The percentage change was then calculated for each element (Ca, P).

### Evaluation of the antibacterial effect of the modified and unmodified primer against *Streptococcus mutans*

#### Well diffusion (agar-based) test

The agar plate surface was inoculated by distributing a volume of the microbial inoculum (*Streptococcus mutans* ATCC 25175) over the whole agar surface. A hole with a diameter of 6 mm was aseptically created using a sterile cork borer, and a volume of 100 mL of the Stela primer modified with HA-NPs and ZnO-NPs, together with the unmodified Stela primer, was put into the well. Agar plates were incubated at 37 °C under hypoxic conditions for *Streptococcus mutans* utilizing a Heracell™ VIOS 160i CO2 Incubator (Thermo Scientific, Germany). The three drugs were incorporated into the agar medium and evaluated for their capacity to suppress the growth of the microbial strain [31].

#### Minimal inhibitory concentration (MIC)

##### Broth Micro dilution preparation

Small volumes of prepared broth (brain heart infusion) [32] were dispensed into sterile, plastic microdilution trays with round or conical bottom wells. Each well contained 0.1 mL of broth. The broth microdilution method described in M07 is the same methodology outlined in ISO 20776-1 [22].

##### Preparing and storing diluted modified Stela primer

Microdilution trays were prepared by performing intermediate two-fold dilutions of the modified Stela primer in broth. A single pipette was used to measure all diluents, and thereafter, the stock modified Stela primer was added to the initial tube. A new pipette was utilized for each successive dilution step. The altered Stela/broth solutions were allocated into plastic microdilution trays utilizing a dispensing apparatus (Multidrop™ Combi, Thermo Scientific, USA). Modified Stela primer dilutions were prepared in a minimum of 10 mL of broth. The dispensing apparatus subsequently administered 0.1 (± 0.02) mL into each of the 96 wells of a standard tray.

##### Inoculum preparation and inoculation of *S. mutans*

The inoculum was prepared by suspending isolated colonies from an 18- to 24-hour agar plate in non-selective medium (Brain Heart Infusion broth agar). The suspension was subsequently modified to attain a turbidity corresponding to a 0.5 McFarland standard. This produced a suspension with approximately 1–2 × 10^8 colony-forming units (CFU)/mL of *S. mutans*. A photometric instrument (McFarland Densitometer DEN-1B, Biosan, Latvia) was used to evaluate the inoculum tube and the 0.5 McFarland standard against a white card with contrasting black lines.

Ideally, within 15 minutes of creating the modified inoculum solution, it was diluted in distilled water, resulting in each well containing roughly 5 × 10^5 CFU/mL (ranging from 2 to 8 × 10^5 CFU/mL). Each well was thereafter infected utilizing a microdilution tray and an inoculator that dispenses a volume not surpassing 10% of the well volume (=10 µL of inoculum in 0.1 mL of antimicrobial agent solution).

##### Incubation

The inoculated microdilution trays were placed in an ambient-air incubator at 35±2°C for 16 to 20 hours, initiated within 15 minutes of inoculum addition. To ensure uniform incubation temperature across all cultures, microdilution trays were limited to a maximum stacking height of 4. To avoid desiccation, each tray was securely enclosed in a plastic bag using plastic tape or a snug plastic cover before incubation.

##### Determining minimal inhibitory concentration end points

The MIC is the minimal concentration of an antimicrobial agent that completely prevents the growth of the organism in the microdilution wells. A pure culture of a designated microbe was cultivated overnight and subsequently diluted in the same broth to a concentration of 1 × 10^5 to 1 × 10^6 cfu/ml. A stock dilution of the antimicrobial test material is prepared at around 100 times the anticipated MIC.

A double-fold dilution (1000-1.95 ug/ml) was prepared in 96-well microtiter plates using Brain Heart Infusion broth. A negative control was included, with serial double-folded dilutions (1000-1.95 µg/mL) prepared without inoculation by the test organism to establish a baseline and avoid turbidity in the tested materials. An aliquot of the positive control was cultured to determine the bacterium's baseline concentration. The microtiter plates were thereafter incubated at the specified temperature and duration (at 35±2°C for 16 to 20 hours).

Turbidity was visually assessed, and Optical Density (OD) was measured at 630 nm using a BioTek 800 TS microplate reader (USA). The growth in wells containing the modified Stela primer was compared with that in the growth-control wells (unmodified Stela primer) used in each test set when determining the growth endpoints [33].

#### The minimum bactericidal concentration test

The MIC dilution method was used to determine the MBC; at least 2 of the more concentrated test product dilutions were plated and enumerated to quantify viable CFU/mL. The MBC represents the minimum concentration required to achieve a specified reduction (e.g., 99.9%) in CFU/mL relative to the MIC dilution. The tested solutions were subsequently compared to Gentamycin 1 mg (Memphis Company for Pharmaceutical & Chemical Industries, Egypt) as a control [34].

### Statistical analysis

Statistical analysis was performed using SPSS 27®, GraphPad Prism®, and Microsoft Excel 2016. Shapiro–Wilk and Kolmogorov–Smirnov (CLSI M07) tests for normality indicated that the data were nonparametric; accordingly, comparisons between groups were performed using the Kruskal–Wallis test. The significance level was set to be at *P* = 0.05.

## Results

### Morphological examination of ZnO nanoparticles

**Fig. 2:**
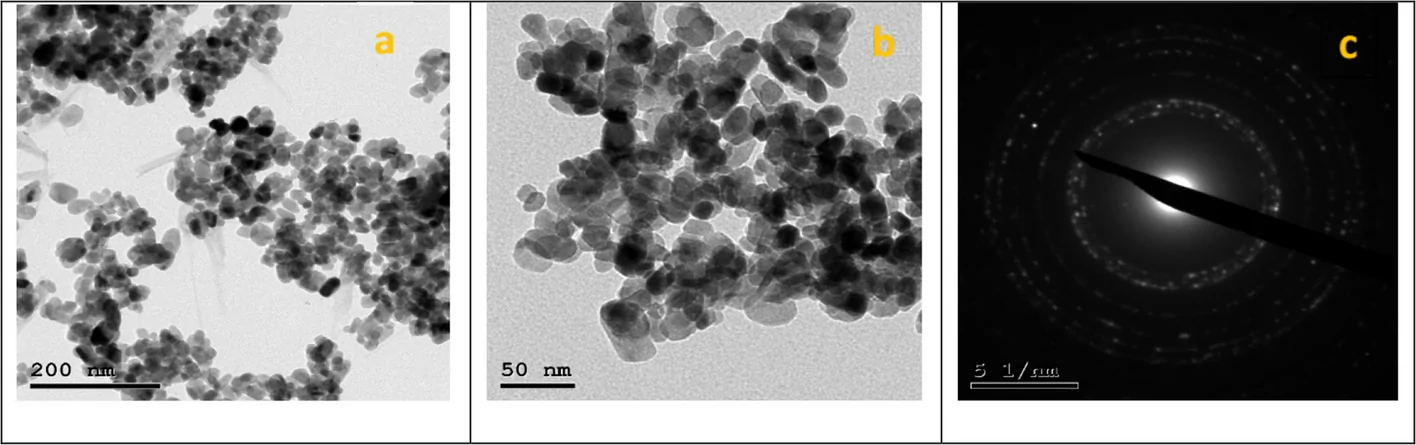
**a-c** Transmission electron microscopy (TEM) for ZnO nanoparticles with a spherical-like shape

TEM analysis of ZnO nanoparticles confirmed the presence of zinc oxide agglomerated nanoparticles in the nanoscale with an average size of 20 ± 5 nm and a spherical-like shape (Fig. 2a, b, c).

### X-ray diffraction (XRD) analysis of ZnO nanoparticles

**Fig. 3:**
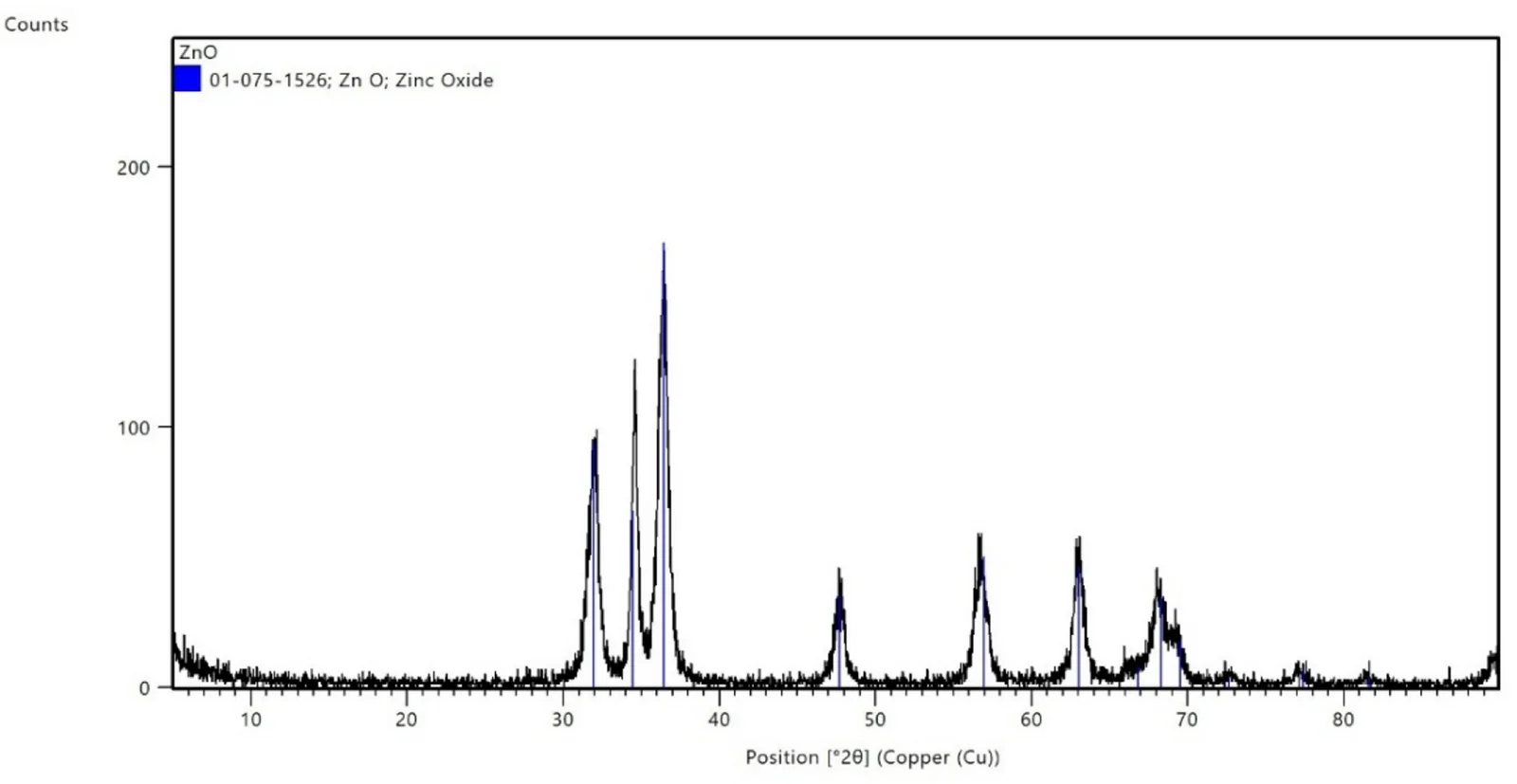
X-ray diffraction (XRD) pattern of ZnO nanoparticles with intense peaks at 31°, 34°, and 36°

Figure 3 revealed a definite line broadening of the XRD peaks, indicating that the prepared material consists of particles in the nanoscale range. The graph clearly shows intense peaks at 31°, 34°, and 36°, confirming the presence of crystalline ZnO nanoparticles with the wurtzite hexagonal structure (JCPDS 36–1451). The sample is highly crystalline and phase-pure.

### Morphological examination of HA nanoparticles

**Fig. 4:**
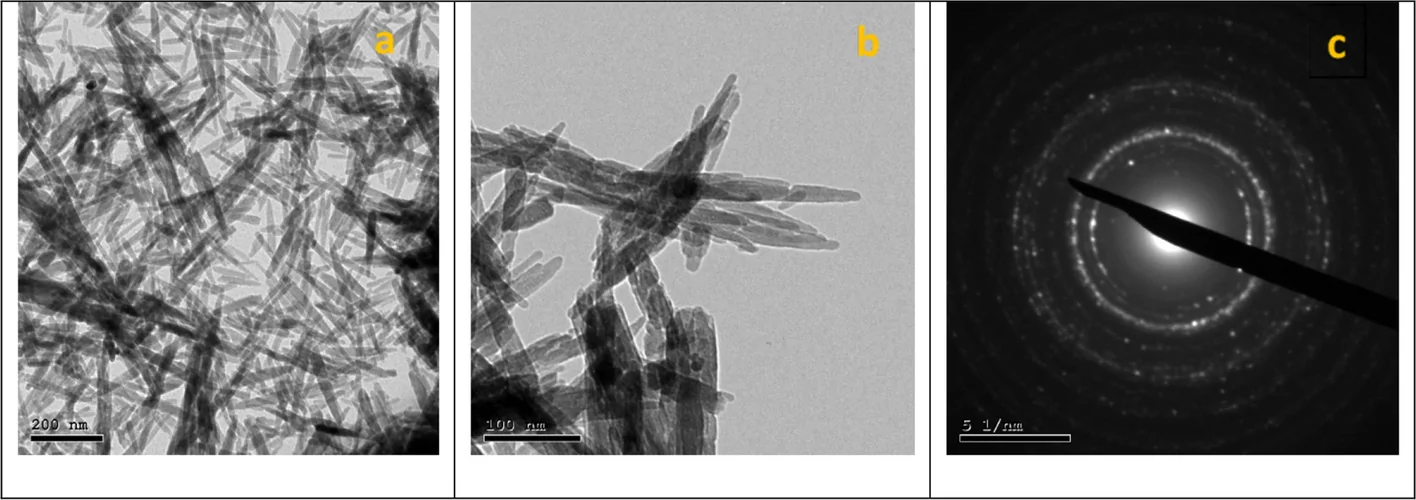
**a-c** Transmission electron microscopy (TEM) for HA nanoparticles with a needle-like shape

TEM analysis of HA nanoparticles confirmed the presence of HA particles at the nanoscale, with needle-like nanoparticles of an average width of 10 ± 5 nm and length of 100 nm ± 10 nm (Fig. 4a, b, c).

### X-ray diffraction (XRD) analysis of HA nanoparticles

**Fig. 5:**
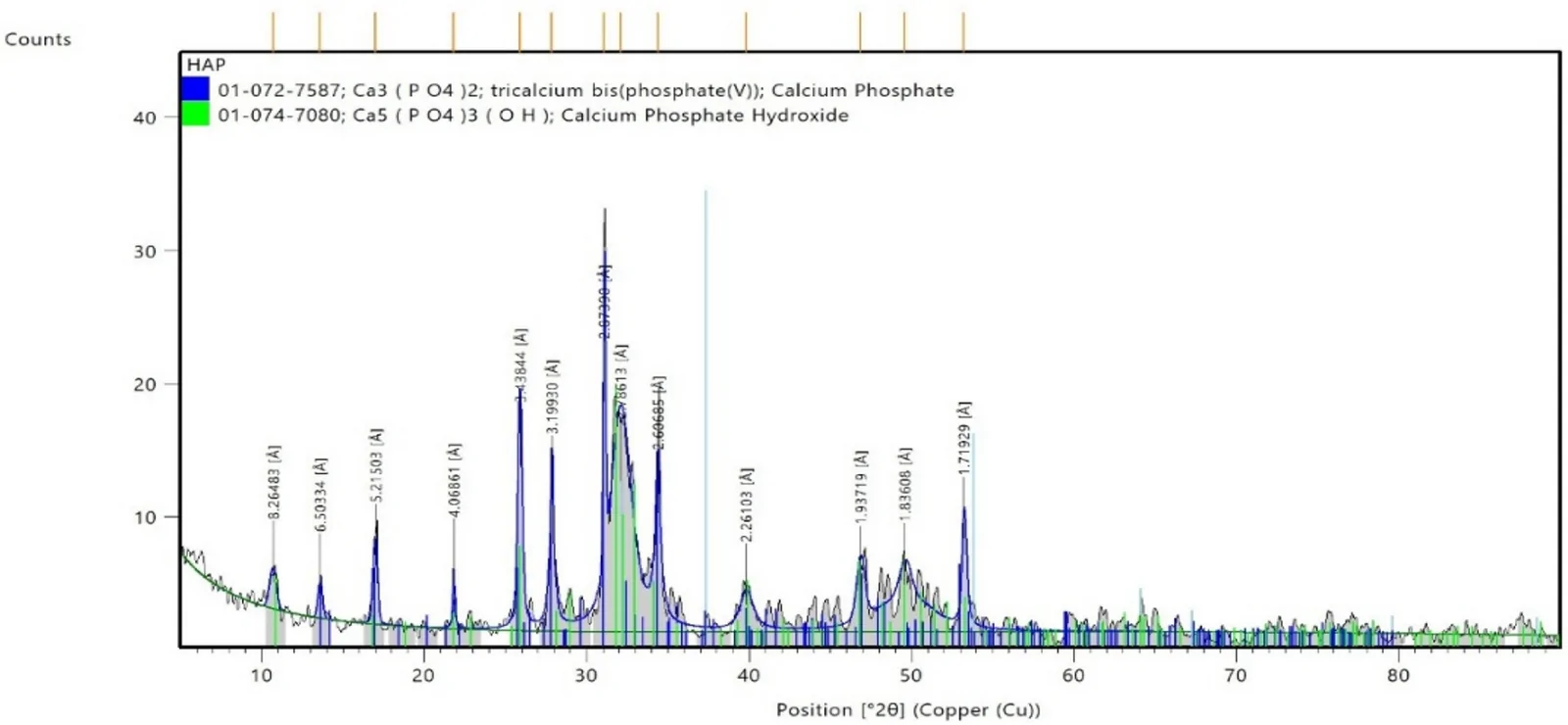
X-ray diffraction (XRD) pattern of HA nanoparticles, with Sharp, well-defined peaks indicating good crystallinity

The pattern shows the main characteristic of HA nanoparticles at peaks (around 25–26°, a strong cluster ~ 31–33°, ~ 39–40°, ~ 46–47°, ~ 49–53°) consistent with hexagonal hydroxyapatite, Ca5(PO4)3(OH) (Fig. 5). The image also shows a second reference to Ca3(PO4)2 (tricalcium phosphate, TCP), indicating partial decomposition or incomplete conversion to pure HA. Sharp, well-defined peaks indicate good crystallinity. Peak broadening indicates nanocrystalline domain size and possibly microstrain.

### Quantitative measurement of microleakage evaluation

#### Intergroup comparison (Comparison between groups)

**Fig. 6:**
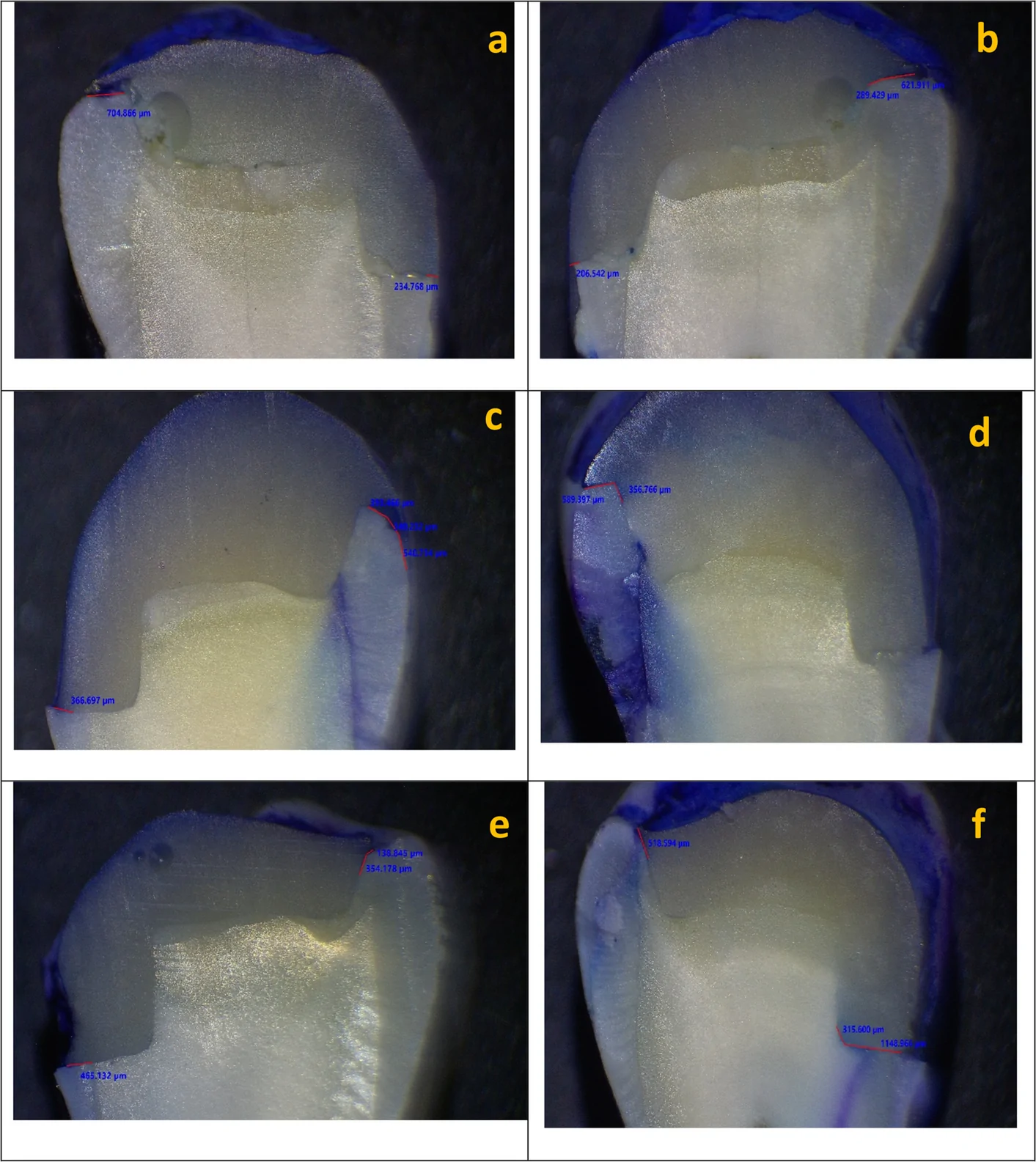
Stereo microscope images of dye penetration for the Group M1 (ZnO-NP) (**a**, **b**), Group M2 (HA-NP) (**c**, **d**), and Group M3 (control) (**e**, **f**) showing microleakage at the restoration margin, gingivally, and occlusally, indicating the extent of marginal dye penetration

Descriptive results of microleakage in all groups regarding occlusal and gingival surfaces were presented in Table 2 and Fig. 6. Comparison between groups was performed by using the Kruskal–Wallis test, which revealed that:

- The mean gingival microleakage values were highest in Group M3 (567.06 ± 404.18) and lowest in Group M2 (437.75 ± 311.72); however, the difference was not statistically significant (*P* = 0.34).
- For the occlusal section, the mean values ranged from 467.11 ± 175.13 (Group M3) to 639.31 ± 413.67 (Group M2), again with no significant difference (*P* = 0.15).
- The overall microleakage means showed a similar trend (Group M1: 580.02, Group M2: 538.53, Group M3: 517.09), with no statistically significant variation among groups (*P* = 0.331).

**Table 2:** Descriptive results of microleakage in all groups regarding all sections

Parameter	Group M 1 (ZnO-NP)		Group M 2 (HA-NP)		Group M 3 (control)		*P* value
	Min/Max (Median)	Mean ± SD	Min/Max (Median)	Mean ± SD	Min/Max (Median)	Mean ± SD	
Gingival	202.54/1037.23 (580.24)	557.11 ± 281.93	0.00/1099.50 (366.70)	437.75 ± 311.72	175.58/1466.53 (465.13)	567.06 ± 404.18	0.34
Occlusal	276.83/912.34 (643.76)	602.94 ± 191.38	0.00/1279.43 (669.01)	639.31 ± 413.67	117.09/775.07 (493.02)	467.11 ± 175.13	0.15
Overall	411.12/842.78 (557.44)	580.02 ± 146.33	205.11/821.06 (504.69)	538.53 ± 200.92	146.34/993.56 (479.08)	517.09 ± 226.27	0.331
*P* value	0.91		0.17		0.51		

#### Intragroup comparison (Comparison between occlusal and gingival)

Descriptive results of microleakage in all groups regarding occlusal and gingival surfaces were presented in Table 2. Comparison between occlusal and gingival surfaces was performed by using the Wilcoxon signed rank test, which revealed that:

- *Group M1*: In Group M1, the mean gingival microleakage value was 557.11 ± 281.93, while the occlusal section recorded a slightly higher mean of 602.94 ± 191.38; the difference between the two sections was not statistically significant (*P* = *0.91*).
- *Group M2*: In Group M2, the gingival microleakage mean was 437.75 ± 311.72, whereas the occlusal section demonstrated a higher mean of 639.31 ± 413.67. The difference did not reach statistical significance (*P* = *0.17*).
- *Group M3*: For Group M3, the mean gingival microleakage was 567.06 ± 404.18, slightly exceeding the occlusal mean of 467.11 ± 175.13. While the gingival section tended to show greater leakage, the difference was not statistically significant (*P* = *0.51*).

### Stereo optical microscope images

Selected Stereo microscope images (Fig. 6) for the three groups showed the cross-section of the prepared sample, the surface morphology, and the layer interface, with the measured microleakage length indicated.

### Elemental analysis results of the tooth/primer interface using energy dispersive X-ray (EDX)

**Fig. 7:**
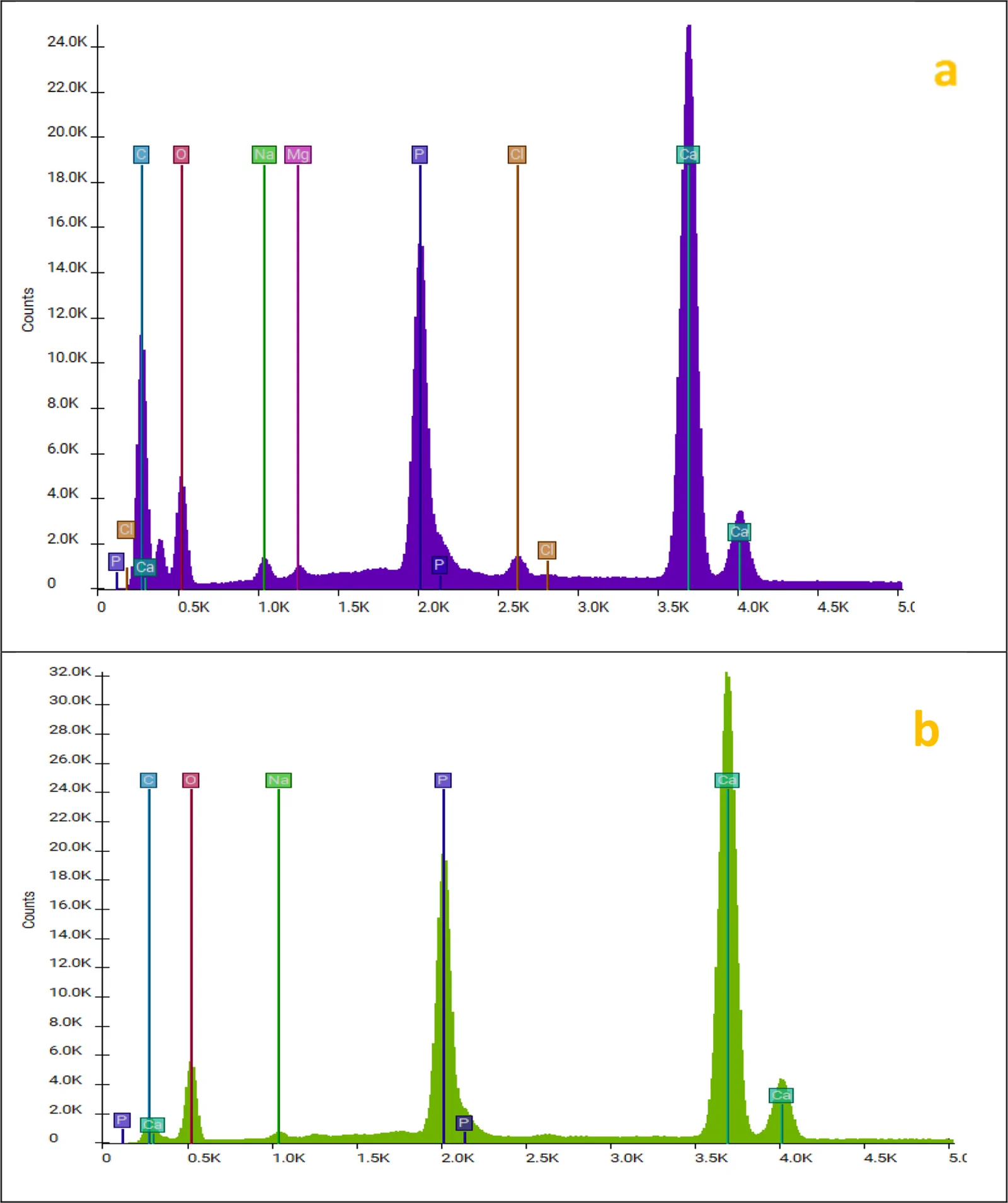
Elemental analysis of calcium and phosphorus levels of Group E1 (ZnO-NP) (**a**) away and (**b**) at the tooth/primer interface

**Fig. 8:**
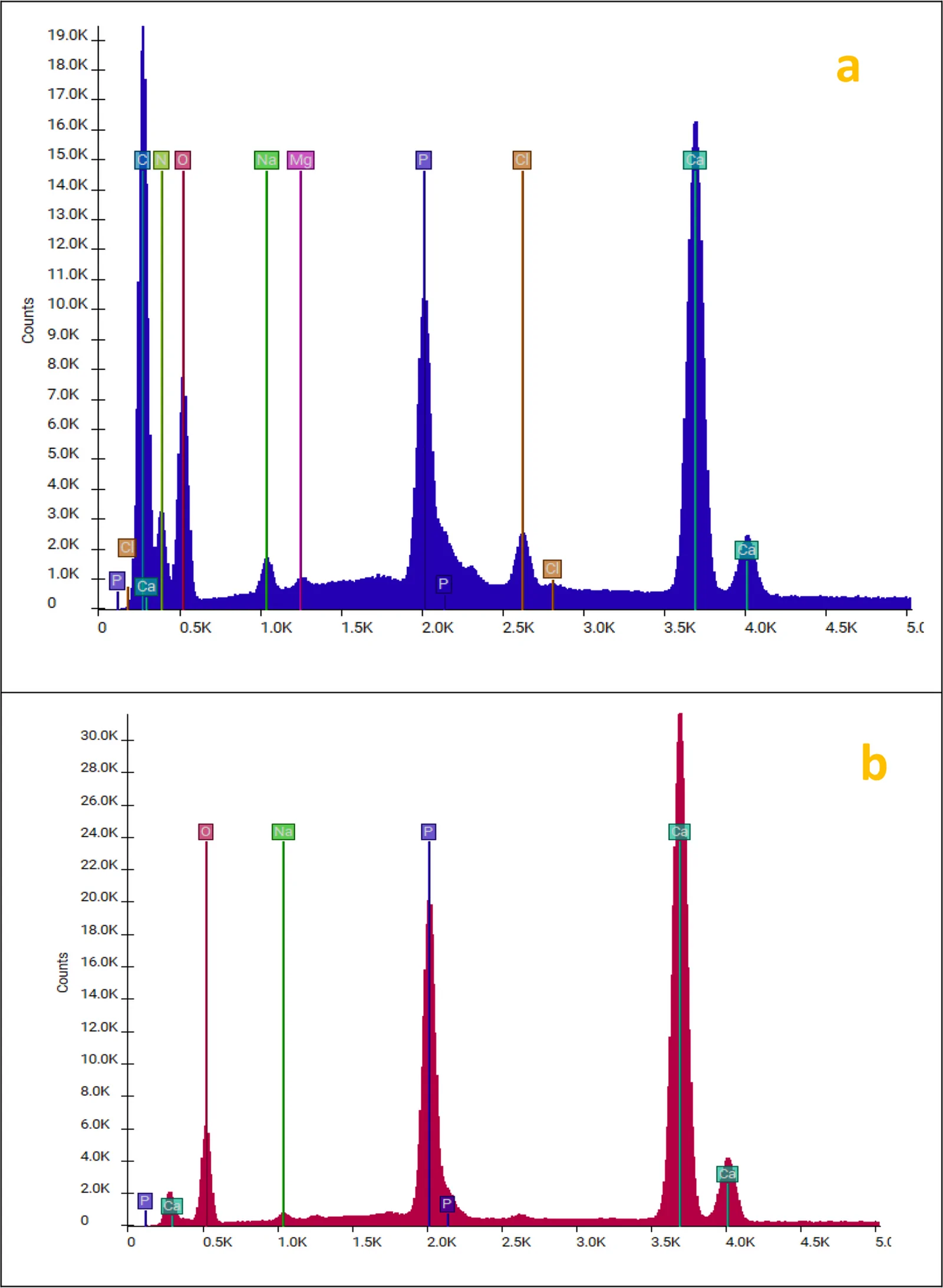
Elemental analysis of calcium and phosphorus levels of Group E2 (HA-NP) (**a**) away and (**b**) at the tooth/primer interface

**Fig. 9:**
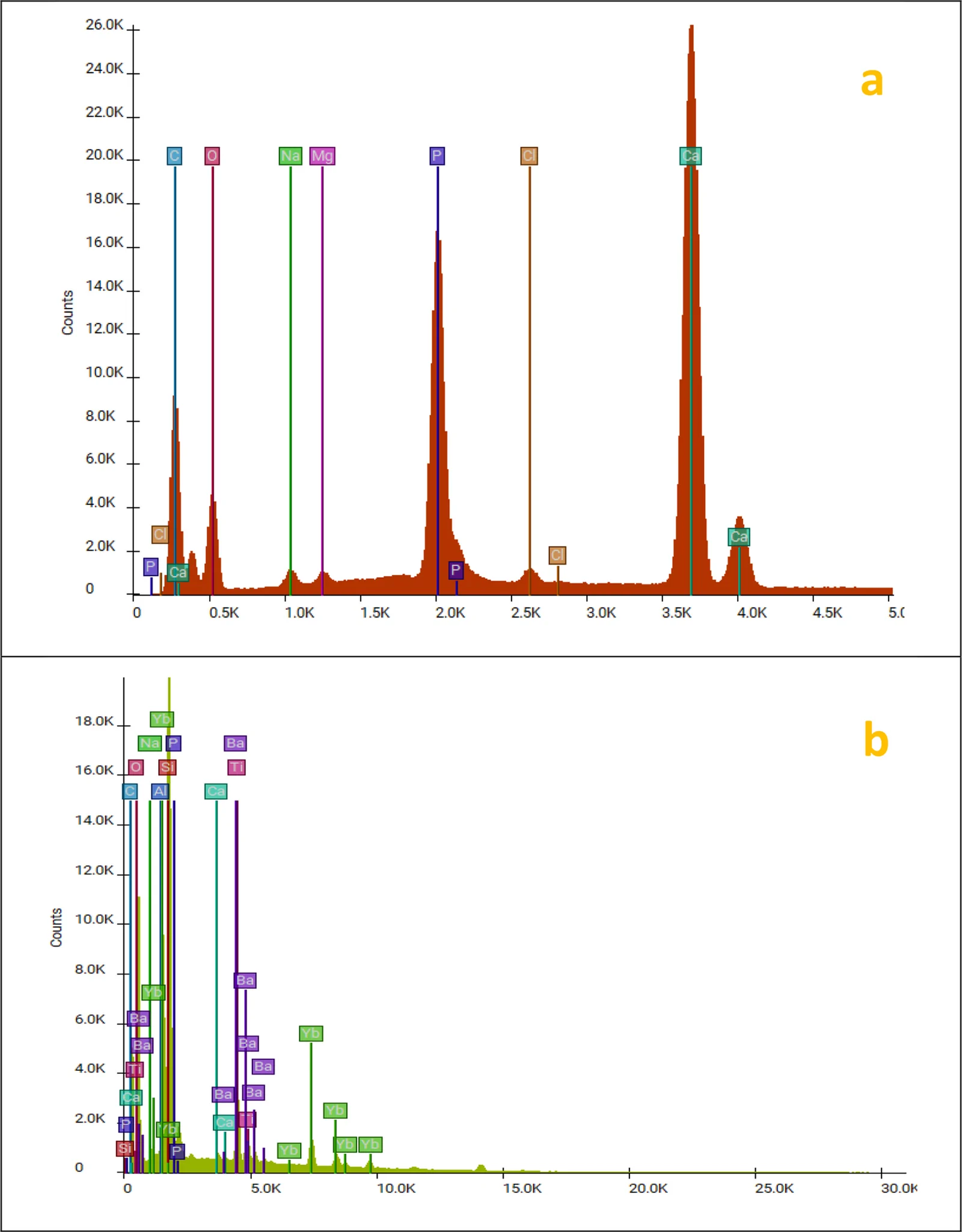
Elemental analysis of calcium and phosphorus levels of Group E3 (control) (**a**) away and (**b**) at the tooth/primer interface

The EDX elemental analysis of Calcium and Phosphorus for each group, away from and at the tooth/primer interface, is shown in Figs. 7, 8, and 9. Regarding Group E3, elemental analysis revealed the presence of other elements, including Silicon, Barium, Yttrium, Aluminum, and Titanium, at the tooth/primer interface (Fig. 9b).

### Comparison of the Mean % change of calcium and phosphorus and Ca/P ratio levels away from and at the tooth/primer interface

Table 3 presents the inter-group comparison of the mean percentage differences in calcium (Ca%), phosphorus (P%), and the Ca/P ratio at the tooth/restoration interface and away from it for groups E1, E2, and E3. Both Group E1 and Group E2 demonstrated a marked increase in Ca% and P% at the interface compared to areas away from the restoration. This increase was statistically significant (*p* < 0.001), indicating superior mineral deposition and interaction with tooth structure. Group E2 showed the highest Ca% (35.46 ± 1.03) and P% (20.55 ± 0.36) at the interface, indicating the most significant remineralizing potential among all groups. Group E1 also exhibited significant mineral gain, but to a lesser extent than Group E2. In contrast, Group E3 showed minimal differences between interface and away measurements for Ca% and P%, with no statistically significant change (*p* > 0.05), suggesting negligible mineral interaction. Regarding the Ca/P ratio, only Group E2 showed a significant increase (*p* < 0.05), whereas Groups E1 and E3 exhibited non-significant changes (*p* > 0.05). These findings indicate that Group E2 material demonstrated the highest bioactivity and mineralizing capability, followed by Group E1, while Group E3 showed the lowest performance.

**Table 3:** Comparison of Mean % difference of calcium and phosphorus and Ca/P ratio levels away from and at the tooth/primer interface

Group	Element	Mean*±*SD (away)	Mean*±*SD (interface)	*P*-value	
Group E1 (ZnO-NP)	Ca%	9,47*±*0.19	29.08*±*4.79	< 0.001	Significant
	P%	5.42*±*0.12	16.39*±*2.97	< 0.001	Significant
	Ca/P ratio	1.75*±*0.03	1.78*±*0.09	> 0.05	Non-Significant
Group E2 (HA-NP)	Ca%	5.30*±*0.95	35.46*±*1.03	< 0.001	Significant
	P%	3.50*±*0.36	20.55*±*0.69	< 0.001	Significant
	Ca/P ratio	1.51*±*0.14	1.73*±*0.07	< 0.05	Significant
Group E3 (control)	Ca%	6.06*±*5.38	6.50*±*5.83	> 0.05	Non-Significant
	P%	3.97*±*2.78	3.89*±*2.61	> 0.05	Non-Significant
	Ca/P ratio	1.29*±* 0.71	1.19*±* 0.61	> 0.05	Non-Significant

### Antibacterial effect results

#### Well diffusion (agar-based) results

**Fig. 10:**
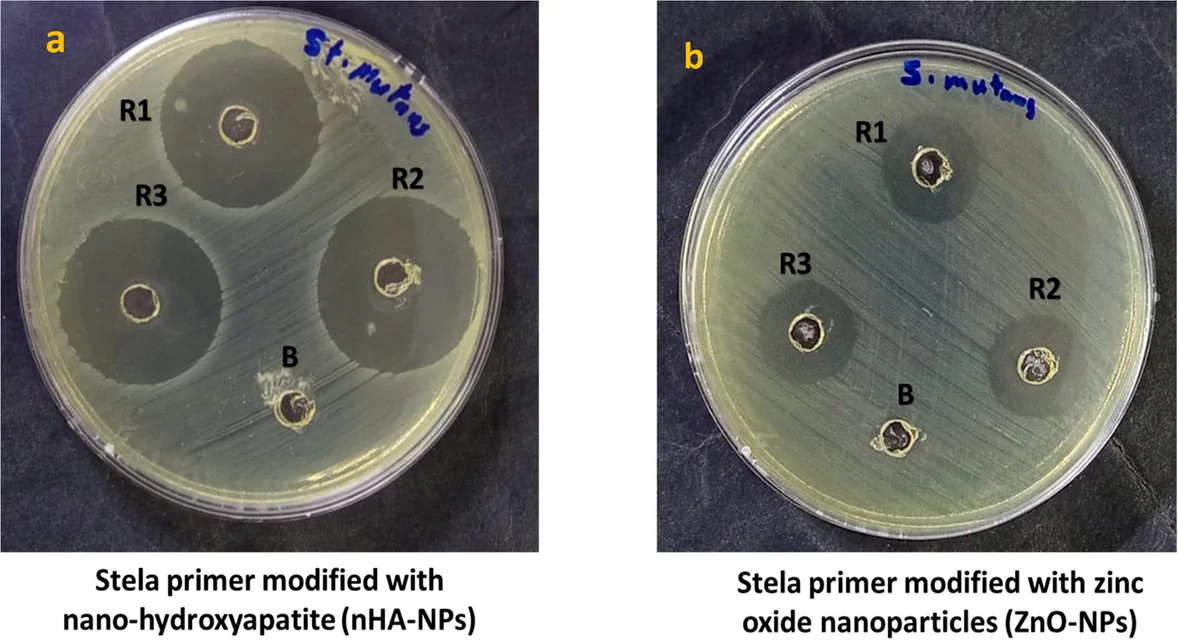
Inhibition zone of (*S. mutans* ATCC 25175) produced by Stela primer modified with HA-NPs (R1, R2, R3) (**a**), Stela primer modified with ZnO-NPs (R1, R2, R3) (**b**), and Stela primer without modification (B) (**a** and **b**)

The antibacterial activities of the modified Stela primer with ZnO and HA nanoparticles, as well as the Stela primer without modification, were tested against S. mutants and quantitatively measured; inhibition zones of more than 6 mm were considered positive. In bacterial cultures, the modified Stela primer with HA nanoparticles showed the largest mean inhibition zone (29.67 mm) compared with the modified Stela primer with ZnO nanoparticles (18.33 mm). In contrast, Stela Primer didn’t produce inhibition zones against *S. mutans* (Table 4 and Fig. 10).

**Table 4:** Inhibition zone of (*Streptococcus mutans* ATCC 25175) by Stela primer modified with ZnO-NPs and Stela primer modified with HA-NPs

*S. mutans ATCC 25175*	Inhibition zone diameter (mm)					
	R1	R2	R3	Mean	SD	SE
Modified primer with ZnO-NPs	18	18	19	18.33	0.58	0.19
Modified primer with HA-NPs	29	31	29	29.67	1.15	0.38

*SD* Standard Deviation, *SE* Standard Error

#### Minimal inhibitory concentration (MIC) and Minimum Bactericidal Concentration (MBC) results

The minimal inhibitory concentration (MIC) and Minimum Bactericidal Concentration (MBC) of both Stela primer modified with ZnO-NPs and Stela primer modified with HA-NPs showed similar results to the 1 mg concentration of Gentamycin (control), regarding the MIC (15.62 µg/mL) and MBC (31.25 µg/mL) (Table 5). However, the unmodified Stela primer showed no antibacterial activity against *S. mutans.*

**Table 5:** Minimal Inhibitory Concentration (MIC) and Minimum Bactericidal Concentration (MBC) of Stela primer modified with ZnO-NPs and Stela primer modified with HA-NPs

Sample	MIC (µg/mL)	MBC (µg/mL)	MBC/MIC Index
Control (1 mg Gentamycin)	15.62	31.25	2
Modified Stela primer with ZnO-NPs	15.62	31.25	2
Modified Stela primer with HA-NPs	15.62	31.25	2

## Discussion

Advancements in resin composite technology have led to the introduction of self-adhesive resin composites, eliminating the need to precondition the tooth structure with dental adhesives. Notwithstanding the efficiency and convenience of self-adhesive resin composites, empirical data are insufficient regarding their adaptability and antibacterial efficacy. Moreover, laboratory evaluations of marginal gap assessment and microleakage quantification may yield a more comprehensive understanding of the sealing efficacy of these adhesives, which has clinical significance.

Therefore, the present study evaluated the effect of incorporating zinc oxide nanoparticles (ZnO-NPs) and hydroxyapatite (HA-NPs) into Stela self-adhesive primer on antibacterial activity against *Streptococcus mutans,* the primary etiologic agent of dental caries, the extent of marginal microleakage in Class II restorations, and elemental analysis of the tooth–primer interface. The null hypothesis was partially rejected because there were statistically significant differences observed in antibacterial activity and mineral content when ZnO-NPs and HA-NPs were incorporated into the Stela primer compared with the unmodified primer. However, there is no difference in marginal microleakage.

A precise marginal seal is a significant goal for direct restorative materials. Inadequate adhesion to dental substrates results in microgaps caused by the polymerization shrinkage of resin composites, promoting bacterial infiltration, recurrent caries, and ultimately, the dislodgment of restorations. Consequently, it was imperative to examine the microleakage of Stela self-adhesive resin composites in class II cavities with occlusal and gingival edges to anticipate the material's performance in clinical settings.

Regarding the microleakage values, the mean gingival microleakage values were highest in Group M3 (567.06 ± 404.18), followed by Group M1 (557.11 ± 281.93), while the lowest value was recorded in Group M2 (437.75 ± 311.72); however, the difference was not statistically significant (*P* = 0.34). For the occlusal section, the mean values ranged from (639.31 ± 413.67) for Group M2, followed by (602.94 ± 191.38) for Group M1, and the least mean value for Group M3 (467.11 ± 175.13), again with no significant difference (*P* = 0.15). The occlusal surface of the restoration is set up closest to the light-curing unit, resulting in minimal energy density loss during curing. Class II restorations present challenges, especially when the cervical margins are deep [35]. If a material shows less microleakage at the gingival margin than at the occlusal margin [36], this is a favorable result, but it doesn't mean the seal is perfect. The material is still exhibiting some level of leakage [37]. The incorporation of HA-NPs has been shown to improve hybrid layer integrity and reduce nanoleakage in specific adhesive systems [11]; this may explain the lowest mean value in the gingival area recorded in Group M2, despite the non-significant difference between all groups.

Other groups (Group M1) and (Group M3) showed comparable microleakage levels. This suggests that the incorporation of ZnO-NPs at 5% w/w did not alter bond formation to enamel and dentin to a degree that significantly improved sealing ability, despite the presence of GDMA, which decreases the polymerization shrinkage of the Stela self-adhesive composite [38]. On the other hand, the improvements in mechanical and antibacterial properties are also dependent on the type of nanofiller being used in the adhesive [39]. TEM analysis of ZnO nanoparticles confirmed the presence of zinc oxide agglomerated nanoparticles, which could increase the viscosity of the Stela primer, thereby contributing to the formation of a microgap at the primer–tooth interface and increasing microleakage [40, 41].

These findings partially agree with Cayo-Rojas et al. [11], who showed that microleakage may remain unaffected when the adhesive's primary performance relies more on polymerization shrinkage behavior and bonding chemistry than on minor compositional adjustments. Furthermore, Stela composite is a chemically cured bulk-fill system, and its slower polymerization rate may have mitigated shrinkage stresses uniformly across all groups [8, 9], explaining the absence of intergroup differences.

EDX analysis provided clear evidence of increased Ca and P deposition at the tooth–primer interface in both experimental groups. This mineral gain was most pronounced in the HA-NP-modified primer (Group E2), which showed the highest Ca% and P% values and the only significant increase in the Ca/P ratio, indicating biomimetic mineral reinforcement of the interface under the conditions of this study.

These findings are consistent with the remineralization potential of hydroxyapatite nanoparticles, which can integrate into the collagen matrix, release ions, and promote apatite precipitation [20, 42]. The Ca/P ratio is often indicative of the mineral phase present. For a healthy, pure hydroxyapatite crystal, the theoretical mass ratio is approximately 2.15. The observed Ca/P ratio is (1.73 *±* 0.07), approaching that of biological hydroxyapatite, suggesting formation of a stable mineral phase capable of enhancing interfacial durability [43].

ZnO-NPs modified primer (Group E1) showed a significant but less pronounced mineral increase, likely attributable to Zn-mediated nucleation phenomena previously described in adhesive systems [18]. In contrast, the unmodified primer (Group E3) showed negligible mineral change, reinforcing that the observed interfacial remineralization was nanoparticle-driven.

Notably, elements such as barium, silicon, and aluminum detected in Group E3 align with the filler composition of the Stela system and indicate passive filler migration rather than active mineral interaction. Incorporating ZnO or HA nanoparticles in Group E1 and E2 may reduce or alter the release of barium, silicon, and aluminum, as increased filler weight can decrease ion release [44]. Another reason could be the change in the direct contact required between the primer and the Stela self-adhesive composite, as the manufacturer mentioned.

Both agar-well diffusion and broth microdilution methods were used to evaluate inhibitory and bactericidal activity. Overall, the findings confirm that nanoparticle-modified primers exhibit superior antibacterial activity compared with unmodified primers. The well diffusion assay demonstrated that both ZnO-NPs and HA-NPs produced inhibition zones against *S. mutans*, but nHA-NPs produced a significantly larger zone (29.67 mm) than ZnO-NPs (18.33 mm). The broader inhibition observed with HA-NPs suggests greater infusibility and more potent antibacterial activity in the agar medium [45]. These results agree with recent studies showing that nanoscale hydroxyapatite exhibits intrinsic antibacterial activity, primarily attributed to its ability to disrupt bacterial cell wall integrity, release calcium and phosphate ions, and alter local pH [46]. The nanoscale size improves surface reactivity and facilitates stronger interactions with bacterial surfaces.

In contrast, ZnO-NPs are well known to generate reactive oxygen species (ROS), such as hydrogen peroxide and superoxide ions, which lead to oxidative damage, membrane leakage, and DNA fragmentation in *S. mutans* [47]. Although ZnO-NPs showed antibacterial activity in the current study, the smaller inhibition zone compared with HA-NPs might be attributed to slower diffusion of ZnO particles in agar or to differences in nanoparticle aggregation behavior, which influence their bioavailability.

Both ZnO-NPs and HA-NPs exhibited identical MIC (15.62 µg/mL) and MBC (31.25 µg/mL) values. The MBC/MIC ratio was 2, indicating that both agents exhibit bactericidal, not just bacteriostatic, activity [48]. This ratio falls below 4, the accepted threshold for distinguishing bactericidal from bacteriostatic agents. The finding that both nanoparticles exhibited equal MIC and MBC values suggests that at lower concentrations, their inhibitory capacities converge, even though their diffusion characteristics differed in the agar-based system [49]. The results align with prior literature indicating that both ZnO and HA nanoparticles exhibit strong, dose-dependent inhibitory effects on oral streptococci.

HA-NPs adhere to bacterial surfaces due to their high surface energy, disrupt cell wall integrity, release Ca^2+^ and PO4^3-^ ions, altering osmotic balance, and interfere with bacterial adherence mechanisms essential for *S. mutans* virulence [50].

Zinc Oxide Nanoparticles (ZnO-NPs) generate Reactive Oxygen Species (ROS), Release Zn^2+^, causing metabolic inhibition, increase membrane permeability, and interfere with bacterial glycolysis [51].

Modifying primer formulations with nanoparticles could be a strategy to reduce microleakage and prevent recurrent caries. The strong antibacterial performance demonstrated by the modified primers suggests that they could effectively inhibit *S. mutans* colonization at the tooth–restoration interface. The HA-NPs may offer additional benefits such as remineralization and biocompatibility [52] while ZnO-NPs provide broad-spectrum antimicrobial action and compatibility with resin-based materials. However, future investigation to better understand the mechanistic behavior of the nanoparticles within the cured resin matrix is recommended.

The integration of ZnO-NPs and HA-NPs into primer formulations offers multiple advantages, such as strong antibacterial activity, particularly with HA-NPs, which may reduce secondary caries risk; enhanced mineral deposition at the interface, improving long-term bond integrity; and absence of adverse effects on marginal sealing, indicating structural compatibility with the parent adhesive system [53]. The HA-modified primer appears the most promising due to its combined antibacterial, remineralizing, and biocompatible properties. This is particularly beneficial for high-risk patients in caries-prone environments. The present study has several limitations. Although the incorporation of ZnO and HA nanoparticles enhanced antibacterial activity and interfacial mineral deposition without adversely affecting marginal sealing, other important material characteristics, including cytotoxicity, biocompatibility, bond strength, degree of conversion, polymerization kinetics, water sorption, solubility, color stability, and long-term aging, were not investigated. These properties are essential for determining the clinical applicability of the modified primer formulations and should be addressed in future studies before clinical translation.

## Conclusion

Within the limitations of this in vitro investigation, the findings indicate that:

1. Incorporating ZnO-NPs and HA-NPs into Stela primer enhances its biological potential; however, they did not significantly alter the sealing ability (microleakage) compared to the control.
2. Both nanoparticle-modified primers exhibited potent and comparable bactericidal activity against *Streptococcus mutans*. At the same time, HA-NPs demonstrated the most pronounced antibacterial effect and the most significant improvement in mineral deposition at the tooth–primer interface.
3. Collectively, these results highlight the promising role of nanoparticle-reinforced primers as a bioactive strategy to suppress microbial activity, enhance interfacial mineralization, and potentially reduce the risk of secondary caries in restorative dentistry.
4. Although the relevant results are effective, long-term clinical trials are necessary to confirm the longevity and effectiveness of these modified primers in a complex oral environment.
5. Although the three raw materials (Stela primer, HA-NPs, ZnO-NPs) are each safe individually, the "new material synthesized from these three components" is recommended to undergo future cytotoxicity studies.

## Data Availability

Data are available upon request. Contact the corresponding author for further details.

## Abbreviations

ZnO: Zinc oxide
HA: Hydroxyapatite
ZnO-NPs: Zinc oxide nanoparticles
HA-NPs: Hydroxyapatite nanoparticles
EDX: Energy-dispersive x-ray spectroscopy
*S.**mutans*: *Streptococcus* *mutans*
GDMA: Glycerol dimethacrylate
YbF3: Ytterbium trifluoride
Al2CaO: Calcium aluminate
MEK: Methyl ethyl ketone
TEM: Transmission electron microscope
XRD: X-ray diffraction
CFU: Colony-forming units
MIC: Minimal inhibitory concentration
OD: Optical density
MBC: Minimum bactericidal concentration
Ca/P: Calcium and phosphorus ratio
ROS: Reactive oxygen species
